# Editorial of the Special Issue: Oncolytic Viruses as a Novel Form of Immunotherapy for Cancer

**DOI:** 10.3390/biomedicines5030052

**Published:** 2017-08-24

**Authors:** Zong Sheng Guo, David L. Bartlett

**Affiliations:** Department of Surgery, University of Pittsburgh School of Medicine, and University of Pittsburgh Cancer Institute, Pittsburgh, PA 15213, USA; bartlettdl@upmc.edu

## 1. Background and Purpose of the Special Issue

Oncolytic viruses (OVs), either occurring naturally or through genetic engineering, can selectively infect, replicate in, and kill cancer cells, while leaving normal cells (almost) unharmed. For many years, most investigators had believed that the therapeutic efficacy of OVs depended mainly on direct viral oncolysis; this approach was named “oncolytic virotherapy”. However, more and more accumulating evidence has shown that post-oncolytic anti-tumor immunity induced by OVs has played a major, and in many cases, essential role in OV-mediated therapy. Thus, OV-mediated cancer therapy has recently been designated as a form of immunotherapy, more specifically “oncolytic immunovirotherapy” [1] or “oncolytic immunotherapy” [2,3,4]. The FDA approved the first-in-class drug T-VEC (Imlygic) for advanced melanoma patients in 2015 [5]. This approval validates this novel approach and further stimulates research and development of this novel class of cancer therapeutics. Against this backdrop, this Special Issue addresses the immunological issues and impact relevant to OVs and the immune tumor microenvironment (TME) and discusses current and future rational strategies to further improve OV-mediated cancer immunotherapy.

In this Special Issue, 14 scientific contributions, including 12 reviews and 2 original research articles, cover these cutting-edge and cross-fertilizing research areas. We divided the articles into four subtopics: (1) The use of OVs as versatile platforms for improved cancer immunotherapy strategies; (2) Specific type of OVs, including adenovirus (AdV), Newcastle disease virus (NDV), and vaccinia virus (VV); (3) Particular cancer types such as thoracic cancer and gastrointestinal malignancies; and (4) The roles of a particular cell type (tumor-associated macrophages), biological pathway (autophagy), and delivery methodology for oncolytic AdV.

## 2. OVs as Versatile Platforms for Improved Cancer Immunotherapy Strategies

Chen, Fong, and colleagues provide an overview of OVs: their history, tumor selectivity, multifaceted mechanisms of action, and a variety of strategies employed to augment OV selectivity and efficacy [6]. They also discuss ongoing clinical trials; two phase III trials are worth special mention: CG0070 (oncolytic adenovirus expressing GM-CSF) for bladder cancer patients with failed bacillus Calmette–Guerin (NCT02365818) and Pexa-Vec (JX-594) for advanced hepatocellular carcinoma (HCC) patients (NCT0256755). This paper also serves as a great general introduction to the other papers in this Special Issue. 

Wan and associates discuss how to utilize OVs as platforms to enhance immunotherapy strategies [7]. They provide an excellent summary of experimental studies that have led to the realization that the immune response is paramount in OV-mediated therapy. Among other topics, they discuss engineering OVs to enhance their immune-stimulatory potential. This has often been conducted by expressing an immunostimulatory cytokine (e.g., GM-CSF, IL-2) or chemokines (such as CCL5) from an OV. Interestingly, another direct approach involves encoding bispecific T-cell engagers into oncolytic viral genomes. The authors also discuss OVs as cancer vaccines. OV infection and induction of immunogenic cell death and subsequent anti-tumor immunity within the tumor was termed in situ vaccination in 1999 [8]. Arming OVs with cytokines (e.g., GM-CSF) or co-expressing tumor-associate antigens (TAAs) have been major improvements for this purpose. In addition, a heterologous prime-boost strategy has been utilized to circumvent the antiviral immune response and obtain robust antitumor immunity [9]. Last, the authors discuss combination approaches to synergize therapeutic effects. These include but are not limited to combinations with adoptive immune cell transfer, immune checkpoint inhibitors, or activators. 

Bourgeois-Daigneault and colleagues continue the theme of using OVs as anti-cancer vaccines [10], discussing several vaccination strategies. Indeed, OV vaccination recruits and activates innate and adaptive immune cells, including antigen-presenting cells that prime the immune response and natural killer cells and cytotoxic T lymphocytes that directly kill tumor cells. These platforms can be further enhanced by engineering the virus with immunostimulatory factors (cytokines and chemokines), immune checkpoint blockade, or inhibitors of immunosuppressive factors (such as prostaglandin E2). They also discuss arming the OV with TAA. This strategy has been shown to work with OVs (vesicular stomatitis virus (VSV) and VV) with multiple TAAs and tumor models. Bourgeois-Daigneault et al. discuss heterologous OV prime-boost strategy, other alternative strategies such as coating viruses to retarget cancer, and the main barrier to vial vaccination strategies, the unavoidable immune response to the virus itself. The authors review several creative strategies to overcome this hurdle.

Lattime and Sharp discuss the use of recombinant poxviruses to modulate the TME for cancer immunotherapy [11]. Their key point is that intratumoral vaccination of a poxvirus encoding TAA and immune stimulatory molecules can modulate the TME, inhibiting the immune inhibitory pathways and drive both local and systemic anti-tumor immunity.

## 3. Representative OVs and Targeted Cancer Types

The second group of articles covers representative types of OVs and selected type of cancers for treatment. Yamamoto and associates review strategies of using oncolytic AdVs as chaperones of immune-stimulatory adjuncts [12]. AdVs possess targeted specificity and can induce a strong immune reaction as a key advantage. They highlight key developments using AdVs expressing GM-CSF, CD40L, IFN-α, and IL-12, and combination strategies with immune checkpoint blockade (CTLA-4, PD1/PD-L1 antibodies) or with CAR-T cells. Schirrmacher reviews the clinical progress of developing NDV as an OV [13]. It is interesting to note that NDV is one of the first viruses to be explored as an OV; the first report of using NDV for human cancer was published in 1964. Three protocols have been used to treat cancer patients with NDV as oncolysate vaccines, an OV, or as NDV-infected autologous tumor cell vaccine with NDV (ATV-NDV). Among the many clinical studies using NDV, one trial that stands out is a prospectively randomized phase II/III trial examining the efficacy of ATV-NDV. The authors of that study noted a significant benefit in terms of long-term metastasis-free survival and overall survival in colon cancer patients treated with ATV-NDV [14]. Altomonte and colleagues discuss strategies of utilizing VSV, a negative-strand RNA virus, as an OV to defeat cancer [15]. One key aspect has been to utilize its potential to elicit immune responses to break immune tolerance in the TME. The authors summarize four strategies: (1) Modifying endogenous viral genes to stimulate the production of interferon induction; (2) expressing immune-stimulatory molecules from the virus to enhance anti-tumor immunity; (3) using OVs as cancer vaccines to stimulate adaptive tumor antigen-specific immune responses; and (4) combinations with adoptive T cell transfer for potentially synergistic therapy. 

The vast majority of oncolytic AdVs, when delivered systemically, are phagocytosed and destroyed by Kupffer cells (macrophages) in the liver. Investigators have been developing various approaches to detarget AdVs to the liver through pharmacological and physical pathways. These approaches may include physical retargeting, physical detargeting, chemical shielding, and modifying the ability of the virus to selectively replicate in certain cancer cells. In an original study, Barry and associates compare physical detargeting via hexon-BAP (8 kDa biotin acceptor protein) modification with so-called post-entry targeting using the tumor-selective replicating E1A mutant dl1101/07. They also tested polyethylene glycol shielding (PEGylation) of the AdV in a Syrian hamster tumor model [16]. The authors found that, when delivered systemically, Ad5-hexon-BAP was more effective than the conditionally-replicating Ad5-dl1101/07 (with mutation in E1A protein). As for PEGylation, the modification blunted the virus-induced production of IL-6, but also reduced oncolytic activity.

Three articles focus on the use of OVs in therapy for specific types of cancer. In the first paper, Patel and Dash review the literature on utilizing various OVs for immunotherapy of thoracic cancers [17]. Many studies have been performed on preclinical models of thoracic cancers, and nine clinical trials are ongoing or will be soon initiated. As pointed out by the authors, major issues remain. These hurdles include, first, that viral delivery remains a thorny issue for some OVs; second, complex interactions between the virus, tumor, and host may dictate therapy outcomes. Warner and Chaurasiya discuss the progress of clinical applications of OVs for colorectal cancer [18]. The most promising OV appears to be Pexa-Vec (JX-594) in colorectal cancer patients [19]. In that study, the virus was administered intravenously once every 14 days at doses of up to 3.0e7 pfu/kg for at least two doses. They did not report dose-limiting toxicities and the trial did not reach the maximum tolerated dose. These authors examined a panel of cytokines in the serum and found some interesting patterns. More interestingly, 10 out of 15 patients (67%) had radiographically stable disease. In phase I/II trials with other OVs, no objective clinical responses were observed. Chaurasiya and Warner also summarize recent studies on combination therapies with immune checkpoint inhibitors or with CAR T cells. In another review, Aha and Bekaii-Saab discuss preclinical and clinical studies with OVs in gastrointestinal malignancies [20]. They discuss preclinical studies using oncolytic AdV, HSV, reovirus, VSV, and VV. On the clinical side, so far the most promising therapy is, again, Pexa-Vec (JX-594) with sorafenib. This combination has been under a phase III trial in patients with HCC. The authors point out two major barriers to effective therapy: immunologic tolerance to cancer-specific antigens, and that the delivery efficacy of intravenous administration is limited by hepatic and splenic sequestration of the virus or pre-existing anti-viral antibodies. 

## 4. Effects of Specific Cell Types, Molecular Pathways, and Combination Strategies on Oncolytic Therapy

Cripe and associates discuss functions of tumor-associated macrophages (TAMs) in oncolytic virotherapy [21]. TAMs can be a “friend” or “foe” depending on cancer type, macrophage phenotype (M1 or M2), and the particular OV used. As the authors summarize, TAMs can either support oncolytic virotherapy through enhancing anti-tumor immunity at the cost of hindering direct oncolysis, or through immunosuppressive protection of virus replication at the cost of hindering anti-tumor immunity. Generally speaking, M1 macrophages may lead to enhanced clearance of the virus, yet they appear to be a ‘friend’ in terms of therapeutic efficacy, as they promote antitumor immunity. In contrast, M2 macrophages tend to be a “foe”, as they enhance tumor growth and immunosuppression. However, exceptions were found in glioblastoma, breast, and pancreatic cancers, in which the opposite effects seemed to occur.

Meng, Ding, and colleagues discuss strategies of targeting autophagy for oncolytic virotherapy [22]. Autophagy has well-established tumor-suppressive properties. Recent studies have concluded that autophagy inhibits cancer development through the orchestration of inflammation and immunity. While attenuating tumor-promoting inflammation, autophagy enhances the processing and presentation of TAAs, thereby stimulating anti-tumor immunity [23]. Some OVs may enhance antitumor immunity by inducing autophagic cell death. The review lists at least eight OVs that can modulate autophagy and thus enhance antitumor immunity. While other OVs may not themselves induce autophagy, combining OV with certain pharmaceutic agents to stimulate or inhibit autophagy is feasible to enhance therapeutic efficacy.

Al-Shammari and colleagues conducted original research on the combination of OV and the chemotherapeutic agent (5-fluorouracil, 5FU) for cancer cells in vitro. The authors show that the combination of a non-pathogenic strain of NDV (LaSota strain) with the commonly used chemotherapeutic agent 5FU has synergistic cytotoxic effects on different cancer cells in vitro [24].

## 5. Challenges and Future Directions of Oncolytic Immunotherapy

Most articles discuss the major hurdles of utilizing a particular type of OV or cancer for OV-mediated therapy. One major hurdle is regarding the low efficiency of delivering OV to tumor tissues and replication throughout the entire tumor. The ability of this approach to achieve consistent therapeutic responses is limited. Another hurdle is the need to develop a broad repertoire of systemic therapeutic immune cells to control not only primary tumors, but also disseminated disease. Antiviral immunity may prematurely clear the OV and limit the development of anti-tumor immunity, thus reducing therapeutic efficacy mediated by the OV. Another obstacle is that OVs possess toxicities when delivered at high doses, just like other classes of drugs, as shown by Imlygic (T-VEC). The solutions to some of these hurdles may be universal or unique to each individual OV. Many more studies are needed to overcome these major issues. Among many strategies to achieve superior efficacy, the combination of OVs with other anti-cancer drugs to act synergistically may be the most promising.

In summary, the articles in this Special Issue provide both basic scientists and clinicians with a comprehensive, up-to-date overview of the rapidly developing field of novel cancer therapy, as well as an in-depth review of the utility of OVs as vehicles for cancer immunotherapy. These articles provide not only the current status, but also the major obstacles of the field and novel strategies to overcome these hurdles in the near future. Although challenges remain, the future of the field looks very bright, as numerous novel combination regimens are expected to join the repertoire of standard care options for cancer patients in the near future [25].
